# Surfactant protein D prevents mucin overproduction in airway goblet cells via SIRPα

**DOI:** 10.1038/s41598-024-52328-5

**Published:** 2024-01-20

**Authors:** Kentaro Hata, Kazuya Tsubouchi, Kunihiro Suzuki, Daisuke Eto, Hiroyuki Ando, Toyoshi Yanagihara, Keiko Kan-o, Isamu Okamoto

**Affiliations:** Department of Respiratory Medicine, Graduate School of Medical Sciences, Kyushu University, Fukuoka, 812-8582 Japan

**Keywords:** Cell biology, Molecular biology, Diseases, Medical research

## Abstract

Mucin overproduction is a common feature of chronic airway diseases such as asthma and chronic obstructive pulmonary disease (COPD), and exacerbates their underlying respiratory condition. Surfactant protein D (SP-D) protects against airway diseases through modulation of immune reactions, but whether it also exerts direct effects on airway epithelial cells has remained unclear. Therefore, we sought to investigate the inhibitory role of SP-D on mucin production in airway epithelial cells. We prepared air–liquid interface (ALI) cultures of human primary bronchial epithelial cells (HBECs), which recapitulated a well-differentiated human airway epithelium. Benzo(a)pyrene (BaP), a key toxicant in cigarette smoke, induced mucin 5AC (MUC5AC) production in ALI-cultured HBECs, airway secretory cell lines, and airway epithelia of mice. Then, the protective effects of SP-D against the BaP-induced mucin overproduction were examined. BaP increased MUC5AC production in ALI cultures of HBECs, and this effect was attenuated by SP-D. SP-D also suppressed the BaP-induced phosphorylation of extracellular signal-regulated kinase (ERK) and MUC5AC expression in NCI-H292 goblet-like cells, but not in NCI-H441 club-like cells. Signal regulatory protein α (SIRPα) was found to be expressed in HBECs and NCI-H292 cells but absent in NCI-H441 cells. In NCI-H292 cells, SP-D activated SH2 domain-containing tyrosine phosphatase-1 (SHP-1), downstream of SIRPα, and knockdown of SIRPα abolished the suppressive effects of SP-D on BaP-induced ERK phosphorylation and MUC5AC production. Consistent with these in vitro findings, intratracheal instillation of SP-D prevented the BaP-induced phosphorylation of ERK and Muc5ac expression in airway epithelial cells in a mouse model. SP-D acts directly on airway epithelial cells to inhibit mucin secretion through ligation of SIRPα and SHP-1-mediated dephosphorylation of ERK. Targeting of SIRPα is therefore a potential new therapeutic approach to suppression of mucin hypersecretion in chronic airway diseases such as COPD and asthma.

## Introduction

Mucin overproduction is a common feature of chronic airway diseases such as asthma and chronic obstructive pulmonary disease (COPD)^1,2^. Pathological mucus contains a relatively high proportion of mucin 5AC (MUC5AC) and therefore has a high viscosity and elasticity that render it difficult to clear^3^. Moreover, air pollution and cigarette smoke, which are rich in polycyclic aromatic hydrocarbons such as benzo(a)pyrene (BaP), promote mucin hypersecretion^4–7^. Such excessive mucus production not only diminishes the quality of life of affected individuals but also exacerbates their underlying respiratory condition^3^. Although common expectorants—such as ambroxol, *N*-acetylcysteine, and carbocysteine—improve mucociliary clearance to some extent, they are capable only of symptom control and do not target the cause of mucus dysregulation. A better understanding of the key molecular pathways that are dysregulated in association with mucin overproduction is therefore needed to provide a basis for the development of new therapeutic strategies^8^.

Surfactant protein D (SP-D), a member of the collectin family of proteins, plays a central role in pulmonary host defense. SP-D is mostly produced by airway club cells and alveolar type 2 cells in the lung, where it mediates the aggregation of inhaled pathogens and allergens as well as regulates immune cells through interaction with collectin receptors^9^. Many studies have demonstrated its protective effects against airway diseases. In individuals with COPD or severe asthma, for example, SP-D leakage from the lung as a result of increased vascular permeability associated with inflammation leads to disease exacerbation^10–12^. However, the possible direct effects of SP-D on airway epithelial cells, in which dysregulation of mucus production contributes to the development and exacerbation of chronic airway diseases, have remained largely unexplored. In the present study, we examined whether SP-D might affect mucin overproduction induced by BaP in air–liquid interface (ALI) cultures of human primary bronchial epithelial cells (HBECs). We then investigated the molecular mechanisms underlying this effect of BaP and its observed inhibition by SP-D in human airway epithelial cell lines. We also confirmed the suppressive effect of SP-D on BaP-induced mucin overproduction in a mouse model.

## Materials and methods

### Reagents and antibodies

Recombinant human SP-D (#1920-SP) and BaP (B1760) were obtained from R&D Systems (Minneapolis, MN) and Sigma-Aldrich (St. Louis, MO), respectively. Antibodies to β-tubulin (#2128; Cell Signaling Technology (CST), Danvers, MA), to MUC5AC (#ab198294; Abcam, Cambridge, UK), to phospho-ERK1/2 (#4370, CST), to ERK1/2 (#9102, CST), to phospho-p38 (#9211, CST), to p38 (#9212, CST), to SIRPα (#13379, CST), to phosphor-SHP-1 (#8849, CST), and to SHP-1 (#3759, CST) as well as horseradish peroxidase-conjugated goat antibodies to rabbit IgG (#7074, CST) were used for immunoblot analysis. Antibodies to MUC5AC (#MA5-12178; Invitrogen, Waltham, MA), uteroglobin (#10490-1-AP; Proteintech, Rosemont, IL), mouse IgG isotype control antibodies (#31903, Invitrogen), rabbit IgG isotype control antibodies (#3900, CST), and goat secondary antibodies conjugated with Alexa Fluor 546 (#A-11030, Invitrogen) or Alexa Fluor 488 (#4412, CST) were used for immunofluorescence microscopy. Antibodies to MUC5AC (#MA5-12178, Invitrogen), phospho-ERK1/2 (#4370, CST), mouse IgG isotype control antibodies (#31903, Invitrogen), and rabbit IgG isotype control antibodies (#3900, CST) were used for immunohistochemistry.

### Cell culture and treatment

HBECs from never-smoker patients with pulmonary nodules and normal lung function were isolated and cultured as described previously^13^. For ALI culture, HBECs between passages 2 and 4 were transferred to collagen-coated Transwell inserts (0.33-cm^2^ polyester filters with a pore size of 0.4 µm; Corning, Corning, NY) at a density of 1.2 × 10^5^ cells/cm^2^ and with an apical volume of 200 μl and basal volume of 500 μl of bronchial epithelial growth medium (BEGM; Lonza, Basel, Switzerland). After the cells had achieved confluence (within 48 h), they were subjected to the ALI condition by removal of the apical medium. They were maintained with 500 μl of PneumaCult-ALI Medium (Stemcell Technologies, Vancouver, Canada) supplemented with heparin solution (Stemcell Technologies) and hydrocortisone stock solution (Stemcell Technologies) in the lower compartment for 28 days to allow differentiation. After ALI culture for 28 days, the cells were exposed (or not) to recombinant human SP-D (10 µg/ml) in 15 µl of PBS at the apical surface for 24 h and were then similarly treated with BaP (100 nM) with or without SP-D (10 µg/ml) three times at 24-h intervals for a total treatment time of 72 h. The cells were then fixed with 10% formalin and embedded in paraffin as described previously^14^ or homogenized for extraction of RNA.

The human airway epithelial cell lines NCI-H292 and NCI-H441 were obtained from ATCC (Manassas, VA) and were maintained in RPMI 1640 medium (Thermo Fisher Scientific, Waltham, MA) supplemented with 10% FBS. These cells were exposed (or not) to recombinant human SP-D (1 µg/ml) for 2 h and then treated with BaP (1 µM) with or without SP-D (1 µg/ml) for 24 h. BEAS-2B cells were maintained in DMEM/F12 medium (Thermo Fisher Scientific) supplemented with 10% FBS. All cell culture was performed at 37 °C under an atmosphere of 5% CO_2_.

### Immunofluorescence analysis

Paraffin-embedded ALI-cultured HBECs were sectioned at a thickness of 5 µm. Sections were depleted of paraffin, autoclaved at 120 °C for 10 min in citrate buffer (pH 6.0) for antigen retrieval, and exposed to a blocking solution (#03649-64; Nacalai Tesque, Kyoto, Japan) for 10 min before incubation overnight in a humidified chamber at 4 °C with primary antibodies or isotype control. Immune complexes were detected by incubation with secondary antibodies for 1 h at room temperature, after which the sections were mounted in mounting medium containing DAPI (#H-1200; Vector Laboratories, Newark, CA). Images were acquired with a BZ-X810 fluorescence microscope (Keyence, Osaka, Japan) and analyzed with Image J software.

### qRT-PCR analysis

Total RNA was extracted from cells with the use of an RNeasy Mini Kit (#74106; Qiagen, Hilden, Germany) and was subjected to RT with a PrimeScript RT Reagent Kit (#RR037; Takara, Kusatsu, Japan). The resulting cDNA was subjected to real-time PCR analysis with SYBR Green PCR Master Mix (#4309155; Thermo Fisher Scientific, Waltham, MA) and the following primers (forward and reverse, respectively): 5′-TGCAGCTATGTGCTGACCAA-3′ and 5′-GCTCAGTGTCACGCTCTTCA-3′ for *MUC5AC*; 5′-TCGGCCACGGAGTTTCTTC-3′ and 5′-GGTCAGCATGTGCCCAATCA-3′ for *CYP1A1*; and 5′-AGGGCTGCTTTTAACTCTGGT-3′ and 5′-CCCCACTTGATTTTGGAGGGA-3′ for *GAPDH*. The relative expression of the target genes was analyzed by the 2^–ΔΔCt^ method and normalized by that of *GAPDH*.

### Apoptosis assay

NCI-H292 and NCI-H441 cells were treated with BaP (10 µM) or DMSO vehicle for 24 h, after which they were stained with the use of Annexin-V-FULUOS Staining kit (#11858777001; Roche Diagnostics Corporation, Indianapolis, IN). Cells were removed using trypsin/EDTA, washed with PBS, and resuspended in 100 μl of Annexin-V-FLUOS labeling solution before incubation for 15 min. Fluorescence was measured by FACSVerse flow cytometer (Becton Dickinson).

### Immunoblot analysis

Cells and lung tissue were lysed in RIPA Lysis Buffer (Fujifilm Wako, Osaka, Japan) supplemented with protease (#P8340, Sigma-Aldrich) and phosphatase (#P0044, Sigma-Aldrich) inhibitors. 20 µg of lysate proteins were loaded on a 6–10% polyacrylamide gel and separated at 120 V for 80 min. Proteins were transferred to a polyvinylidene difluoride membrane at 20 V overnight by using a wet-transfer system. Membranes were blocked in Blocking One solution (#03953, Nacalai Tesque) for 30 min and cut around the predicted molecular weight before incubation first overnight at 4 °C with primary antibodies and then for 1 h at room temperature with secondary antibodies. Immune complexes were detected with ECL reagents (Bio-Rad, Hercules, CA) and a ChemiDoc Touch Imaging System (Bio-Rad).

### Measurement of ROS

NCI-H292 cells were transferred to 96-well black plates (#165305, Thermo Fisher Scientific), treated with SP-D and BaP, washed with PBS, and then exposed to CellROX Deep Red Reagent (#C10422, Invitrogen) for 30 min in a dark CO_2_ incubator at 37 °C. The cells were washed twice with PBS and submerged in 100 µl of RPMI 1640 medium for measurement of fluorescence intensity with an Ensight microplate reader (Perkin Elmer, Waltham, MA).

### RNA interference

NCI-H292 cells were transiently transfected with 5 nM *SIRPA* siRNA (Silencer siRNA, #AM51331; Ambion, Carlsbad, CA) or 5 nM negative control siRNA (Silencer Negative Control No. 1 siRNA, #AM4611; Ambion) with the use of Lipofectamine RNAiMAX Reagent (#13778150, Invitrogen) for 24 h before experiments.

### Animal experiments

All animal experiments (approval number: A19-021-2) were carried out under the Kyushu University Guidelines for Animal Experiments and the ARRIVE guidelines. Eight-week-old female C57BL/6J mice were obtained from Japan SLC (Shizuoka, Japan) and were housed under specific pathogen-free conditions for 1 week before administration of recombinant human SP-D (5 μg in 50 μl of PBS) and BaP (1 mg in 50 μl of tricaprylin) by intratracheal instillation. The mice were sacrificed and analyzed 48 h later. At intratracheal instillation and terminal procedure, the mice were anesthetized with a mixture of medetomidine hydrochloride (0.3 mg/kg), midazolam (4 mg/kg) and butorphanol tartrate (5 mg/kg) intraperitoneally. The mice were monitored for a surgical plane of anesthesia determined by loss of reflexes (lack of response to toe and tail pinch), muscle relaxation, and deep, rhythmic breathing. At termination, the mice were euthanized by exsanguination. The right lung lobes were fixed by intratracheal instillation of 10% neutral-buffered formalin at a pressure of 20 cm H_2_O and paraffine-embedded for histology, then the left lung lobes were taken for protein extraction.

### Immunohistochemistry

Paraffin-embedded mouse lung tissue was sectioned at a thickness of 3 µm. Deparaffinized/rehydrated sections were autoclaved at 120 °C for 10 min in citrate buffer (pH 6.0) for antigen retrieval. Sections were blocked for endogenous peroxidase activity using 3% hydrogen peroxide in methanol for 10 min and exposed to a blocking solution (#03649-64, Nacalai Tesque) for 10 min before incubation overnight in a humidified chamber at 4 °C with primary antibodies or isotype control. Immune complexes were detected by incubation with Simple Stain MAX-PO (M) (#424131; Nichirei, Tokyo, Japan) or Simple Stain MAX-PO (R) (#414341, Nichirei) at room temperature for 1 h before applying chromogen detection using diaminobenzidine (#425011, DAB substrate kit, Nichirei). Counterstaining was performed with Mayer’s hematoxylin (#30002; Muto pure chemicals, Tokyo, Japan) before dehydration. Sections were clarified with xylene and mounted using Marinol (#4197193, Muto pure chemicals). Images were acquired with a BZ-X810 microscope (Keyence) and analyzed with Image J software.

### Statistical analysis

Quantitative data are presented as means ± SEM. Data were compared between two groups with Student’s two-tailed unpaired *t* test and among more than two groups by one-way analysis of variance (ANOVA) followed by Sidak’s multiple comparison test. Statistical analysis was performed with the use of GraphPad Prism 9 (GraphPad Software, San Diego, CA), and a *P* value of < 0.05 was considered statistically significant.

### Ethics approval and consent to participate

All procedures with human samples were approved by the Kyushu University Institutional Review Board for Clinical Research (2022–56), and all patients provided written informed consent in accordance with the principles of the Declaration of Helsinki. All mouse experiments were approved by the Kyushu University Animal Care and Use Committee (A19-021-2).

## Results

### SP-D attenuates BaP-induced MUC5AC production in ALI-cultured HBECs

**Figure 1:**
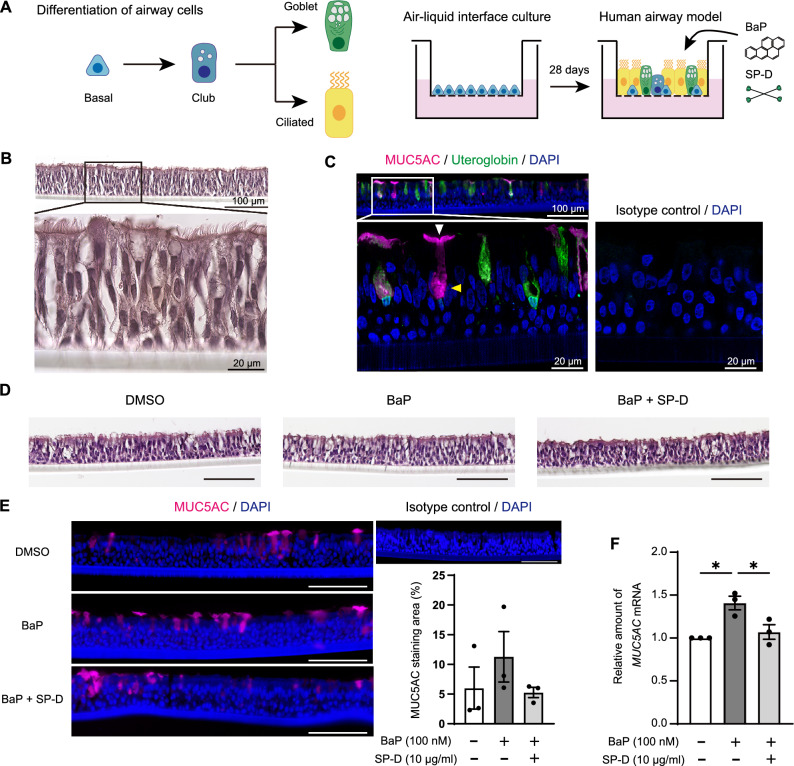
BaP-induced MUC5AC production and its inhibition by SP-D in ALI-cultured human HBECs. (**A**) Major lineage hierarchy of airway epithelial cells and experimental design for ALI culture. HBECs were cultured under the ALI condition until fully differentiated (for 28 days) and were then exposed (or not) to recombinant human SP-D (10 µg/ml) at the apical surface for 24 h before three consecutive applications of BaP (100 nM) with or without SP-D (10 µg/ml) at 24-h intervals. The images were generated using Adobe Illustrator (version 27.9, Adobe Inc., San Jose, CA). (**B**) Hematoxylin–eosin (H&E) staining showing a well-ciliated epithelial surface for HBECs maintained in ALI culture for 28 days. (**C**) Immunofluorescence staining of the fully differentiated ALI-cultured cells. Club and goblet cells were detected with antibodies to uteroglobin and to MUC5AC, respectively. Nuclei were stained with 4′,6-diamidino-2-phenylindole (DAPI). The white and yellow arrowheads indicate mucin secretion onto the epithelial surface and mucin production in secretory cells, respectively. (**D**) H&E staining of the ALI-cultured cells after treatment with DMSO vehicle or with BaP in the absence or presence of SP-D. Scale bars, 100 µm. (**E**) Representative immunofluorescence staining of MUC5AC (scale bars, 100 µm) as well as the area of MUC5AC staining (as a percentage of the area of the airway epithelium) determined from such images for ALI-cultured cells treated with BaP and SP-D as indicated. Quantitative data are means ± SEM (*n* = 3) for ALI cultures established from three independent patients, with 12 areas per culture being calculated and averaged. (**F**) qRT-PCR analysis of *MUC5AC* mRNA abundance in ALI cultures treated with BaP and SP-D as indicated*.* Data are means ± SEM (*n* = 3) for ALI cultures established from three independent patients. *P < 0.05 (one-way ANOVA followed by Sidak’s test).

To model BaP-induced mucin overproduction in the human airway epithelium, we prepared mature mucociliary ALI cultures derived from HBECs, which allow recapitulation of the cell differentiation present in native airway tissues^15^ and the in vivo pattern of exposure of airway epithelial cells to toxic substances (Fig. 1A). The ALI cultures manifested differentiation of HBECs into a pseudostratified epithelium with cilia over 28 days (Fig. 1B). Immunofluorescence staining revealed the presence of airway secretory cell types (uteroglobin^+^ club cells and MUC5AC^+^ goblet cells) as well as mucin secretion onto the epithelium surface (Fig. 1C). First, to determine the optimal concentration of BaP, ALI-cultured cells were treated with BaP at concentrations of 100 nM, 1 μM, and 10 μM. Microscopically observable cell death was noticed with the 1 μM and 10 μM treatments. As a result, the treatment with 100 nM BaP was identified as the optimal concentration. The cells were then treated with BaP (100 nM) for 24 h or 72 h (three applications to the apical side at 24-h intervals). The mRNA expression levels of *MUC5AC* were increased at 24 h following BaP stimulation and further elevated at 72 h (Supplementary Fig. 1). The 72-h BaP treatment increased the area of MUC5AC immunofluorescence staining without altering the morphology of the epithelial surface (Fig. 1D, E).

To investigate the potential effect of SP-D on BaP-induced MUC5AC production in ALI-cultured HBECs, we exposed the cells to recombinant human SP-D (10 µg/ml) for 24 h before treatment with BaP (100 nM) in the continued presence of SP-D. SP-D prevented the stimulatory effect of BaP on MUC5AC production (Fig. 1E). Quantitative reverse transcription and polymerase chain reaction (qRT-PCR) analysis also showed that SP-D significantly attenuated the BaP-induced increase in *MUC5AC* expression (Fig. 1F).

### SP-D inhibits BaP-induced MUC5AC expression and ERK phosphorylation in goblet cells

To examine the ability of SP-D to inhibit BaP-induced MUC5AC production in different airway secretory cell types, we used NCI-H292 and NCI-H441 cells as substitutes for goblet and club cells, respectively^5,16^. The induction of MUC5AC expression in airway epithelial cells by BaP involves the activation of the cytochrome P450 family 1 subfamily A polypeptide 1 (CYP1A1), reactive oxygen species (ROS), as well as extracellular signal-regulated kinase (ERK) and p38 mitogen-activated protein kinase (MAPK) signaling pathways^5,17, 18^. Initially, we investigated alterations in these signaling pathways following BaP stimulation in NCI-H292 cells, focusing on different time points and concentrations. Treatment with BaP (1 µM) induced ERK phosphorylation at 2, 6, and 24 h post-treatment, along with p38 phosphorylation specifically at the 24-h mark (Supplementary Fig. 2). Furthermore, the increase in *CYP1A1* mRNA plateaued at 1 µM after stimulation with 100 nM, 1 µM, and 10 µM of BaP for 24 h, despite a similar elevation in *MUC5AC* mRNA at these doses of BaP (Supplementary Fig. 3A and 3B). Consequently, we selected the conditions of 1 µM BaP for 24 h, as this effectively triggered the BaP-induced signaling pathway.

**Figure 2:**
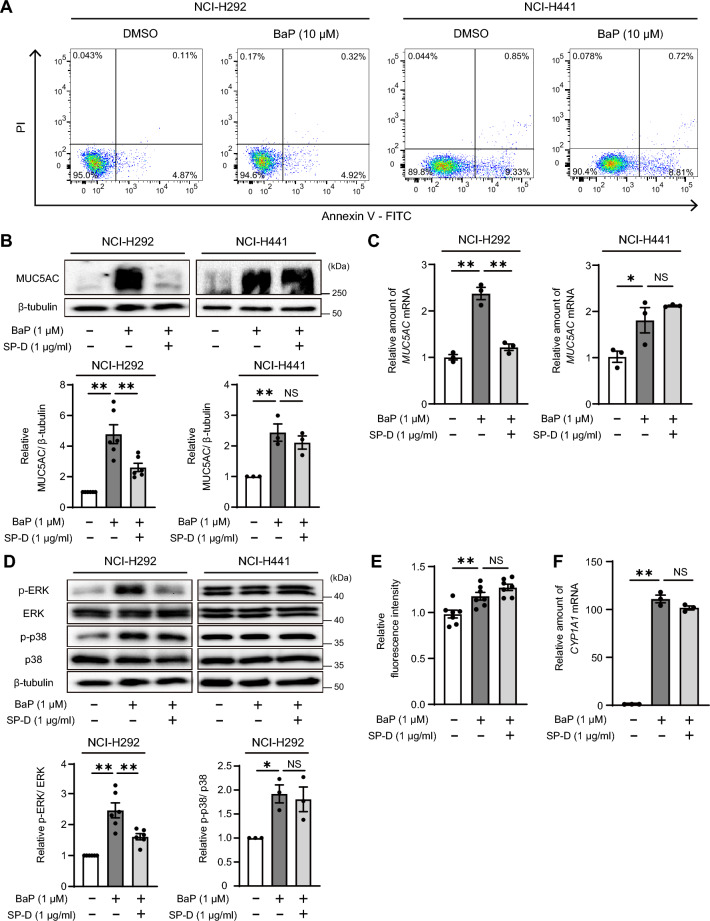
Attenuation by SP-D of BaP-induced MUC5AC expression and ERK phosphorylation in NCI-H292 cells. (**A**) NCI-H292 and NCI-H441 cells were treated with BaP (10 µM) or DMSO vehicle for 24 h, after which they were stained with FITC-labeled Annexin V and propidium iodide (PI) and analyzed by flow cytometry for the detection of necrotic/late apoptotic (FITC-Annexin V and PI double-positive) and early apoptotic (FITC-Annexin V single positive) cells. (**B**) NCI-H292 and NCI-H441 cells were incubated with or without SP-D (1 µg/ml) for 2 h before incubation in the additional absence or presence of BaP (1 µM) for 24 h, and cell lysates were then subjected to immunoblot analysis with antibodies to MUC5AC and to β-tubulin (loading control). Representative blots and quantitative data (means ± SEM) for the MUC5AC/β-tubulin densitometric ratio from six (NCI-H292) or three (NCI-H441) independent experiments are shown. (**C**) Cells treated as in (**B**) were subjected to qRT-PCR analysis of *MUC5AC* mRNA abundance. Shown are representative data (means ± SEM) from three independent experiments. (**D**) Cells treated as in (**B**) were subjected to immunoblot analysis of phosphorylated (p-) and total forms of ERK and p38 MAPK. Quantitative data for the p-ERK/ERK or p-p38/p38 densitometric ratio in NCI-H292 cells are means ± SEM from six or three independent experiments, respectively. (**E**) NCI-H292 cells treated as in (**B**) were assayed for ROS with a quantitative fluorescence assay. Data are means ± SEM from seven independent experiments. (**F**) NCI-H292 cells treated as in (**B**) were subjected to qRT-PCR analysis of *CYP1A1* mRNA abundance. Shown are representative data (means ± SEM) from three independent experiments. *P < 0.05; **P < 0.01, NS (one-way ANOVA followed by Sidak’s test). A representative cropped western blot is shown with the uncropped and multiple exposure images viewable in Additional File.

Subsequent analysis revealed that exposure to1µM BaP increased MUC5AC protein expression as well as mRNA levels (as revealed by immunoblot and qRT-PCR analyses, respectively) without inducing cell death (as revealed by flow cytometric analysis of propidium iodide and Annexin V staining) in both NCI-H292 and NCI-H441 cells (Fig. 2A–C). Following this, cells were exposed to BaP (1 µM) with or without recombinant human SP-D (1 µg/ml) for 24 h after exposure (or not) to SP-D for 2 h. We confirmed that 1 µg/ml of SP-D alone did not affect the mRNA expression of *MUC5AC* and *CYP1A1* (Supplementary Fig. 4). SP-D suppressed BaP-induced MUC5AC expression in NCI-H292 cells but not in NCI-H441 cells (Fig. 2B, C). Moreover, SP-D inhibited the BaP-induced phosphorylation of ERK in NCI-H292 cells, but not that of p38 (Fig. 2D). In NCI-H441 cells, BaP failed to induce phosphorylation of ERK and p38 at any time points (Supplementary Fig. 2 and Fig. 2D). We also found that BaP induced *CYP1A1* expression and ROS production in NCI-H292 cells (Fig. 2E, F). However, SP-D did not suppress these effects of BaP (Fig. 2E, F). Together, our results thus indicated that SP-D attenuates the BaP-induced phosphorylation of ERK in a manner independent of the CYP1A1-ROS pathway, resulting in suppression of MUC5AC expression, in NCI-H292 cells.

### The SIRPα–SHP-1 pathway mediates suppression of BaP-induced ERK phosphorylation and MUC5AC expression by SP-D in goblet cells

**Figure 3:**
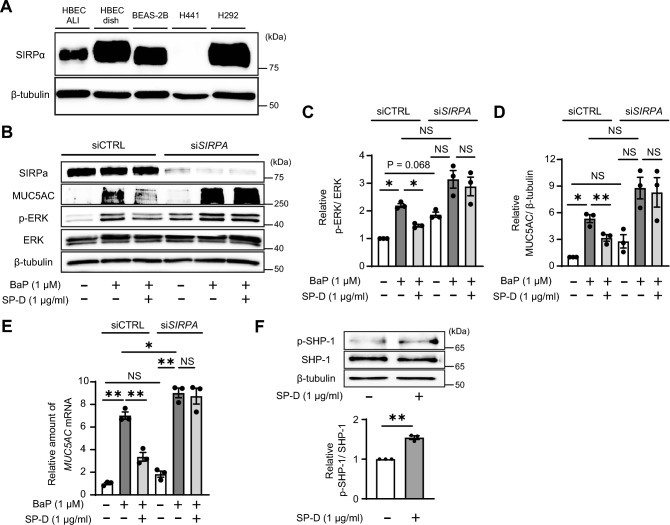
Prevention of ERK phosphorylation and MUC5AC expression by the SIRPα–SHP-1 pathway in NCI-H292 cells. (**A**) Immunoblot analysis of SIRPα in ALI-cultured and dish-cultured HBECs, BEAS-2B cells, NCI-H441 cells, and NCI-H292 cells. (**B**) NCI-H292 cells were transfected with control (siCTRL) or *SIRPA* (si*SIRPA*) siRNAs, incubated first with or without SP-D (1 µg/ml) for 2 h and then in the additional absence or presence of BaP (1 µM) for 24 h, lysed, and subjected to immunoblot analysis with antibodies to SIRPα, to MUC5AC, and to phosphorylated or total forms of ERK. Quantitative data for the (**C**) p-ERK/ERK or (**D**) MUC5AC/β-tubulin densitometric ratio are means ± SEM from three independent experiments. (**E**) NCI-H292 cells treated as in (**B**) were subjected to qRT-PCR analysis of *MUC5AC* mRNA abundance. Shown are representative data (means ± SEM) from three independent experiments. (**F**) Immunoblot analysis of total and phosphorylated forms of SHP-1 in NCI-H292 cells treated with SP-D (1 µg/ml) for 30 min. Quantitative data for the p-SHP-1/SHP-1 densitometric ratio are means ± SEM from three independent experiments. *P < 0.05; **P < 0.01, or NS by one-way ANOVA followed by Sidak’s test (**C**–**E**) or by Student’s *t* test (**F**). A representative cropped western blot is shown with the uncropped and multiple exposure images viewable in Additional File.

To investigate the mechanism underlying the suppressive effect of SP-D on BaP-induced MUC5AC expression in NCI-H292 cells, we examined the possible role of signal regulatory protein α (SIRPα), a plasma membrane protein that is activated by binding to SP-D^19^. Immunoblot analysis revealed that, among human airway epithelial cells, SIRPα is expressed in ALI-cultured HBECs, basal-like dish-cultured HBECs and BEAS-2B cells, and goblet-like NCI-H292 cells, but not in club-like NCI-H441 cells (Fig. 3A). We therefore transfected NCI-H292 cells with a small interfering RNA (siRNA) that targets *SIRPA* mRNA. Knockdown of SIRPα abolished the suppressive effects of SP-D on BaP-induced ERK phosphorylation (Fig. 3B, C) as well as MUC5AC expression at both protein and mRNA levels (Fig. 3B, D, E).

SH2 domain-containing tyrosine phosphatase-1 (SHP-1) is recruited by SIRPα and phosphorylated during stimulation of macrophages with SP-D, and phosphorylated SHP-1 in turn mediates MAPK dephosphorylation^20,21^. We found that SP-D also induced phosphorylation of SHP-1 in NCI-H292 cells (Fig. 3F), suggesting that the SIRPα–SHP-1 pathway is activated by SP-D and that it might contribute to the dephosphorylation of ERK in goblet cells as well as in immune cells. Collectively, our results thus revealed the expression of SIRPα in airway epithelial cells, and they suggested that SP-D activates SIRPα–SHP-1 signaling in NCI-H292 cells and thereby suppresses BaP-induced ERK phosphorylation and MUC5AC expression.

### Intratracheal instillation of SP-D prevents BaP-induced ERK phosphorylation and Muc5ac production in mouse lung

**Figure 4:**
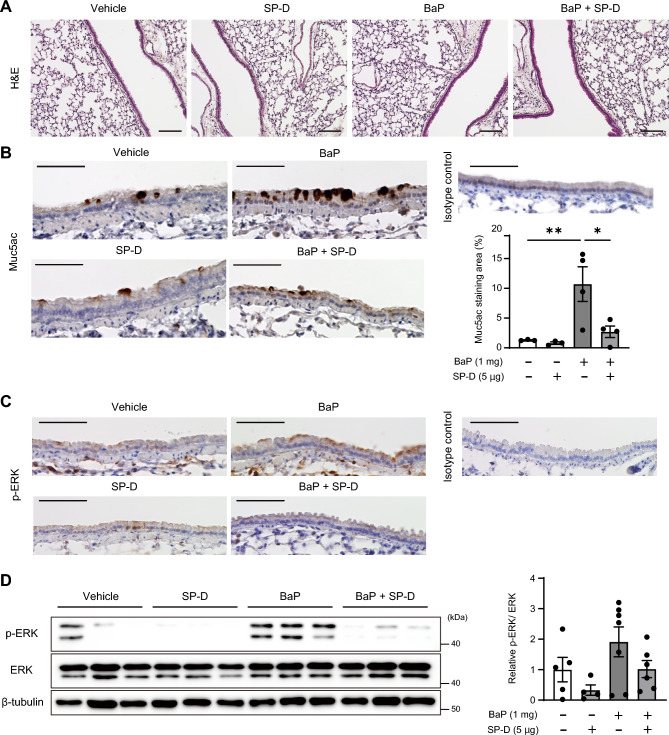
Prevention of ERK phosphorylation and Muc5ac production in mouse airways by intratracheal instillation of SP-D. (**A**) H&E staining of the lung parenchyma and proximal airways of mice at 48 h after intratracheal instillation of vehicle, SP-D (5 µg), or BaP (1 mg) as indicated. Scale bars, 100 µm. (**B**) Representative immunohistochemical staining of Muc5ac in proximal airways of mice treated as in (**A**) and quantitation of the area of Muc5ac staining as a percentage of the airway epithelium area. Quantitative data are means ± SEM from three or four mice. Scale bars, 50 µm. (**C**) Immunohistochemical staining of phosphorylated ERK in proximal airways of mice treated as in (**A**). Scale bars, 100 µm. (**D**) Immunoblot analysis of phosphorylated and total forms of ERK in lung homogenates of mice treated as in (**A**). Representative blots are shown for three mice of each group, and quantitative data for the p-ERK/ERK densitometric ratio are means ± SEM from four to seven mice. *P < 0.05; **P < 0.01 (one-way ANOVA followed by Sidak’s test). A representative cropped western blot is shown with the uncropped and multiple exposure images viewable in Additional File.

To study BaP-induced mucin overproduction in mouse airways, we administered BaP (1 mg in 50 μl of tricaprylin) to C57BL/6J mice by intratracheal instillation and examined the animals 48 h later. Histological examination of lung sections revealed that BaP did not induce morphological changes in the airways or lung parenchyma (Fig. 4A). However, immunohistochemical analysis showed that BaP increased the area of Muc5ac staining in the proximal bronchi (Fig. 4B) and induced ERK phosphorylation in airway epithelial cells (Fig. 4C). The BaP-induced phosphorylation of ERK was also confirmed by immunoblot analysis of lung homogenates (Fig. 4D). To examine whether SP-D might suppress these effects of BaP in the mouse model, we administered SP-D (5 μg in 50 μl of PBS) together with BaP by intratracheal instillation. Consistent with our in vitro results, SP-D indeed attenuated BaP-induced Muc5ac production and ERK phosphorylation in mouse lung (Fig. 4B–D).

## Discussion

**Figure 5:**
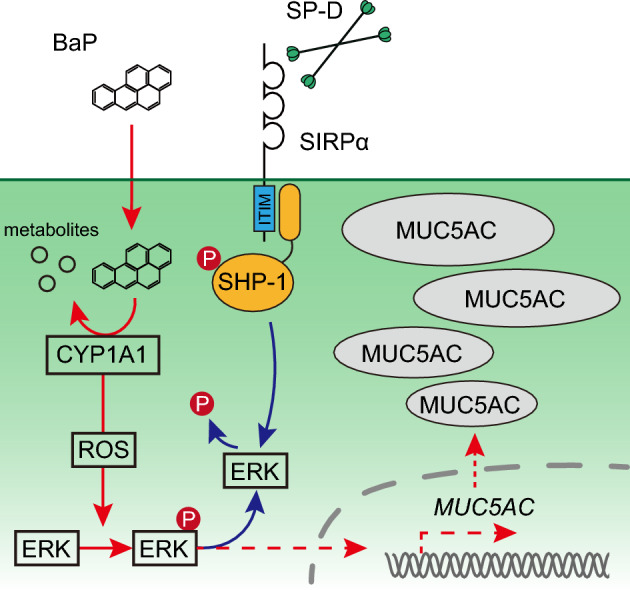
Model for the suppressive effect of SP-D on BaP-induced MUC5AC expression in goblet cells. BaP is metabolized by CYP1A1, and the ROS generated by such metabolism induce ERK phosphorylation and consequent expression of MUC5AC. Ligation of SIRPα by SP-D triggers the phosphorylation of SHP-1, and phosphorylated SHP-1 in turn mediates the dephosphorylation of ERK. The BaP-induced phosphorylation of ERK and consequent MUC5AC expression are therefore suppressed by SP-D. The images were generated using Adobe Illustrator (version 27.9).

In this study, we induced mucin overproduction in airway epithelial cells with BaP and revealed that SP-D suppresses this effect of BaP in goblet cells. We found that SIRPα is expressed in goblet cells and that its ligation with SP-D elicits the phosphorylation of SHP-1, with phosphorylated SHP-1 in turn likely mediating the dephosphorylation of ERK. The BaP-induced phosphorylation of ERK and subsequent expression of MUC5AC are thus attenuated by SP-D in goblet cells (Fig. 5). Intratracheal instillation of SP-D also prevented the BaP-induced phosphorylation of ERK and MUC5AC production in the airways of a mouse model.

SP-D binds to inhaled pathogens and allergens in the lung and prevents their invasion through the airway epithelium surface^22,23^. In addition, SP-D exerts regulatory effects on immune cells, suppressing inflammatory reactions to bacterial or viral challenge as well as T helper 2 responses to allergen exposure^24–28^. These inhibitory effects of SP-D on immune reactions restrain the progression of chronic airway diseases and consequent excessive mucus secretion^9–12^. A Mendelian randomization study showed that genetically elevated serum levels of SP-D were associated with reduced rates of COPD incidence and progression^29^. Our data now show that BaP increased *CYP1A1* expression and ROS production in airway epithelial cells even in the presence of SP-D, indicating that SP-D did not prevent the entry of BaP into airway epithelial cells via direct binding to BaP. Furthermore, we found that SP-D suppressed BaP-induced MUC5AC production in a manner dependent on SIRPα in cultures composed only of airway epithelial cells (ALI-cultured HBECs or dish-cultured NCI-H292 cells). These results thus uncovered a direct and protective effect of SP-D on airway epithelial cells in addition to its roles in the clearance of pathogens or allergens and regulation of immune reactions.

SIRPα is a transmembrane protein expressed predominantly in myeloid cells. It contains immunoreceptor tyrosine-based inhibitory motifs (ITIMs) that serve as a docking site for SHP-1 and thereby facilitate SHP-1 phosphorylation and activation^19^. Activated SHP-1 dephosphorylates key signaling molecules including MAPKs, the kinase Akt, and NF-κB and STAT transcription factors, and thereby modulates the function of myeloid cells^20,21^. SIRPα is activated by binding to SP-D and SP-A, and it attenuates both phagocytosis and the production of pro-inflammatory mediators in macrophages^20,30^. Our results now show that SIRPα was also activated by SP-D in airway epithelial cells, in which it suppressed the BaP-induced phosphorylation of ERK and MUC5AC production. Reflecting a difference in the level of SIRPα expression, these inhibitory effects of SP-D were apparent in goblet-like but not club-like cell lines. Single-cell RNA-sequencing analysis previously revealed cell type-specific gene expression in the lung^31^, and, consistent with our results, mining of these sequencing data revealed *SIRPA* to be expressed in airway epithelial cells^32,33^. We found that siRNA-mediated knockdown of SIRPα tended to increase ERK phosphorylation even in the absence of BaP in NCI-H292 cells, suggesting that SIRPα functions constitutively to regulate the ERK pathway, which plays a key role in goblet cell differentiation and lung remodeling^5,33, 34^. The physiological functions of SIRPα in each airway epithelial cell remain unclear, and further study is required.

Goblet cell metaplasia and mucin overproduction are hallmarks of airway diseases such as asthma and COPD^1,2^. Air pollution and cigarette smoke, which contain high levels of polycyclic aromatic hydrocarbons such as BaP, also stimulate mucin hypersecretion and exacerbate respiratory disease^4–7^. Mucus hypersecretion and plugging cause airway obstruction in chronic airway disorders and thereby increase morbidity and mortality^35^. A better understanding of the key molecular pathways underlying mucin hypersecretion is therefore expected to inform the development of new therapeutic strategies^8^. Endogenous stimuli such as interleukin-13 and epidermal growth factor induce mucin production^36,37^, whereas the WNT coreceptor RYK and the transcription factors YAP and TAZ negatively regulate goblet cell hyperplasia and mucus hypersecretion during lung homeostasis and repair^38,39^. We have now shown that the SIRPα–SHP-1 axis also functions as a negative regulator of mucin overproduction in goblet cells and that exogenous SP-D suppressed BaP-induced ERK phosphorylation and MUC5AC production in a mouse model as well as in cultured airway epithelial cells. Treatment targeting the SIRPα–SHP-1 axis, such as administration of exogenous recombinant SP-D, might therefore be expected to suppress mucin overproduction in individuals with COPD or asthma.

This study has several limitations. First, PneumaCult is utilized in the ALI medium for HBECs, which may influence the expression of MUC5AC to some extent. Nevertheless, consistent results have been validated across distinct experimental systems employing different cell lines and mice. Second, while intracellular mucin was quantified, the analysis did not extend to the measurement of secreted mucus. Third, we utilized HBECs from only three donors.

In conclusion, our data provide new insight into the protective function of SP-D and its target SIRPα in airway epithelial cells. SP-D inhibited BaP-induced phosphorylation of ERK and MUC5AC expression in cultured airway goblet cells through activation of the SIRPα–SHP-1 axis. In addition, intratracheal instillation of SP-D prevented BaP-induced ERK phosphorylation and MUC5AC production in the airways of a mouse model. Activation of the SIRPα–SHP-1 axis in airway epithelial cells is therefore a potential new therapeutic strategy for prevention of mucin hypersecretion.

### Supplementary Information

Supplementary Information 1.

Supplementary Information 2.

## Data Availability

Our experimental datasets used during the current study are available from the corresponding author on reasonable request.

## Abbreviations

COPD: Chronic obstructive pulmonary disease
MUC5AC: Mucin 5AC
BaP: Benzo(a)pyrene
SP-D: Surfactant protein D
ALI: Air–liquid interface
HBEC: Human primary bronchial epithelial cell
ERK: Extracellular signal-regulated kinase
MAPK: Mitogen-activated protein kinase
CYP1A1: Cytochrome P450 family 1 subfamily A polypeptide 1
ROS: Reactive oxygen species
SIRPα: Signal regulatory protein α
siRNA: Small interfering RNA
SHP-1: SH2 domain-containing tyrosine phosphatase-1
