# Supplementary material for: Surfactant protein D prevents mucin overproduction in airway goblet cells via SIRPα
Source: Sci Rep. 2024 Jan 20;14:1799. doi: 10.1038/s41598-024-52328-5 (PMC10799941; doi:10.1038/s41598-024-52328-5)
Supplement: Supplementary file 1 — Supplementary Information 1. [file 41598_2024_52328_MOESM1_ESM.docx]

**Supplementary File**

**Supplementary Figure 1. Time-course *MUC5AC* mRNA expression in ALI-cultured HBECs.**


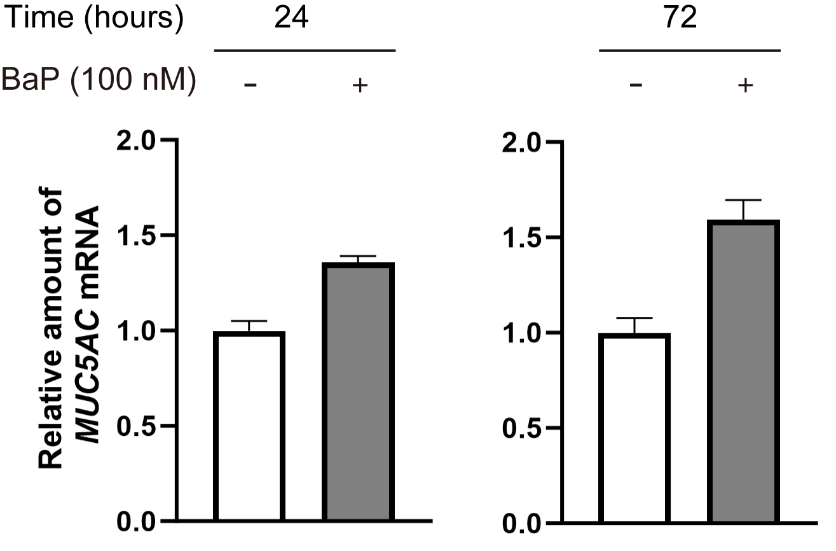
qRT-PCR analysis of *MUC5AC* mRNA abundance in ALI cultures treated with BaP (100nM) for 24 hours and 72 hours*.* Shown are representative data (means ± SEM) for ALI cultures established from a patient.

**Supplementary Figure 2. Time-course immunoblot analysis of phosphorylated (p-) and total forms of ERK and p38 MAPK in NCI-H292 and NCI-H441 cells.**

NCI-H292 and NCI-H441 cells were treated with 1µM of BaP or DMSO vehicle for 2 h, 6 h, and 24 h, after which cells were subjected to immunoblot analysis of phosphorylated (p-) and total forms of ERK and p38 MAPK.


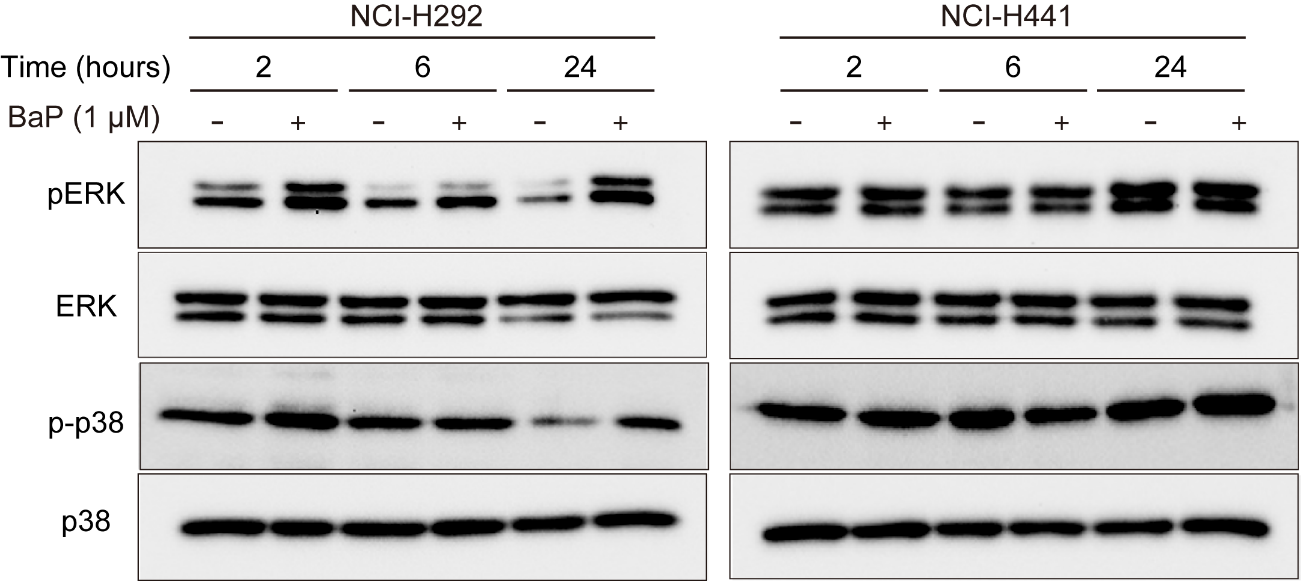


**Supplementary Figure 3. BaP dose-dependent expression of *CYP1A1* and *MUC5AC* mRNA expression in NCI-H292 cells.**


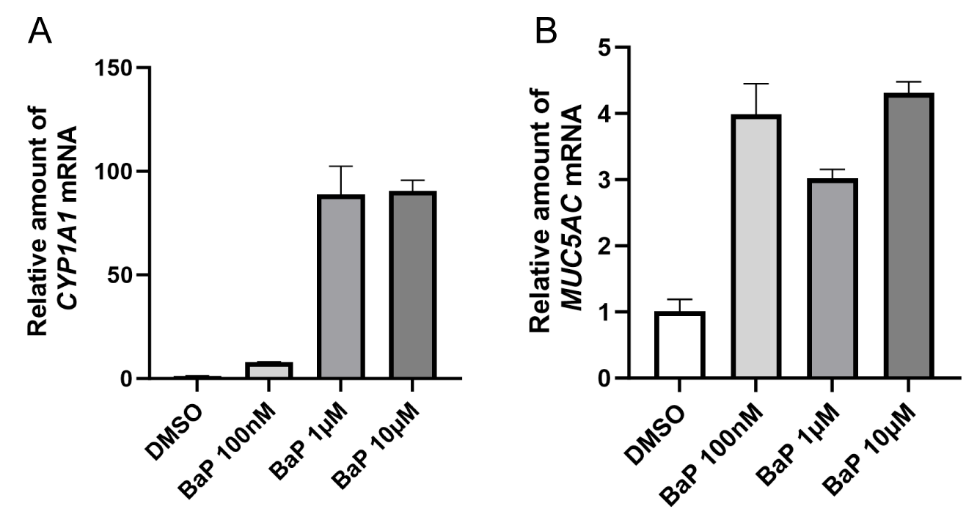
NCI-H292 cells were treated with 100nM, 1µM, and 10µM of BaP or DMSO vehicle for 24 h, after which cells were subjected to qRT-PCR analysis of *CYP1A1* mRNA abundance (*A*) and *MUC5AC* mRNA abundance (*B*). Data are shown as means ± SEM.

**Supplementary Figure 4. Effect of SP-D alone on expression of *MUC5AC* and *CYP1A1* mRNA expression in NCI-H292 cells.**


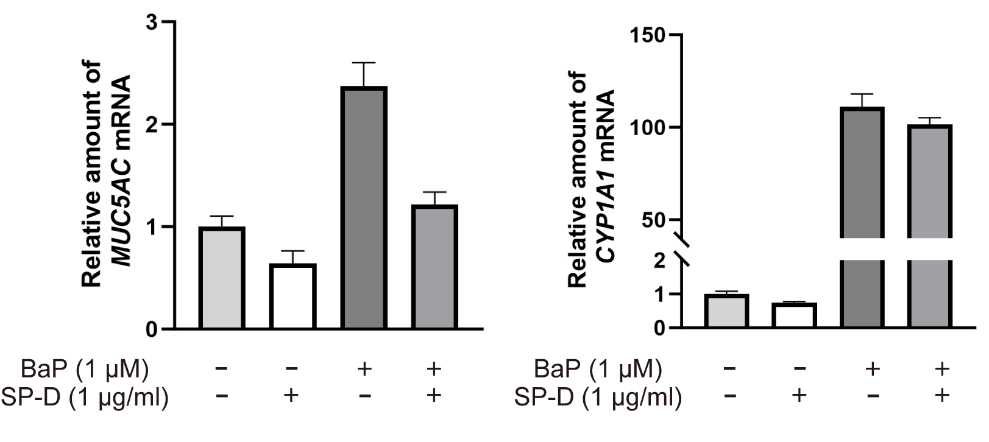
NCI-H292 cells were incubated with or without SP-D (1 µg/ml) for 2 h before incubation in the additional absence or presence of BaP (1 µM) for 24 h, after which cells were subjected to qRT-PCR analysis of *MUC5AC* mRNA abundance and *CYP1A1* mRNA abundance. Data are shown as means ± SEM.
