# Supplementary material for: Surfactant protein D prevents mucin overproduction in airway goblet cells via SIRPα
Source: Sci Rep. 2024 Jan 20;14:1799. doi: 10.1038/s41598-024-52328-5 (PMC10799941; doi:10.1038/s41598-024-52328-5)

**Additional File**

**Uncropped images from Western blots.**

Uncropped Western blot images are shown that correspond to Figure 2B (a), Figure 2D (b), Figure 3A (c), Figure 3B (d), Figure 3F (e), and Figure 4D (f). Dot lines indicate a cropped area.


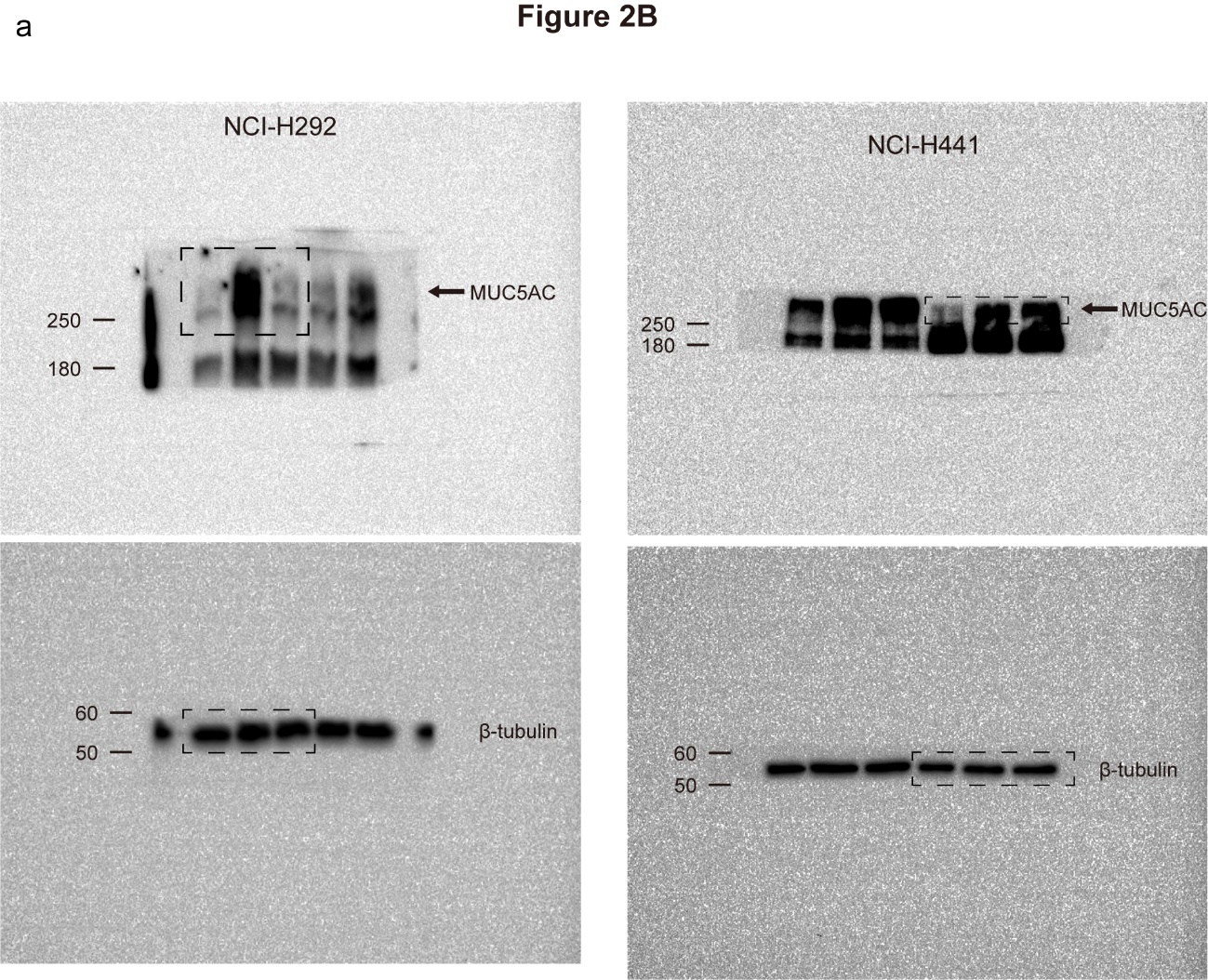


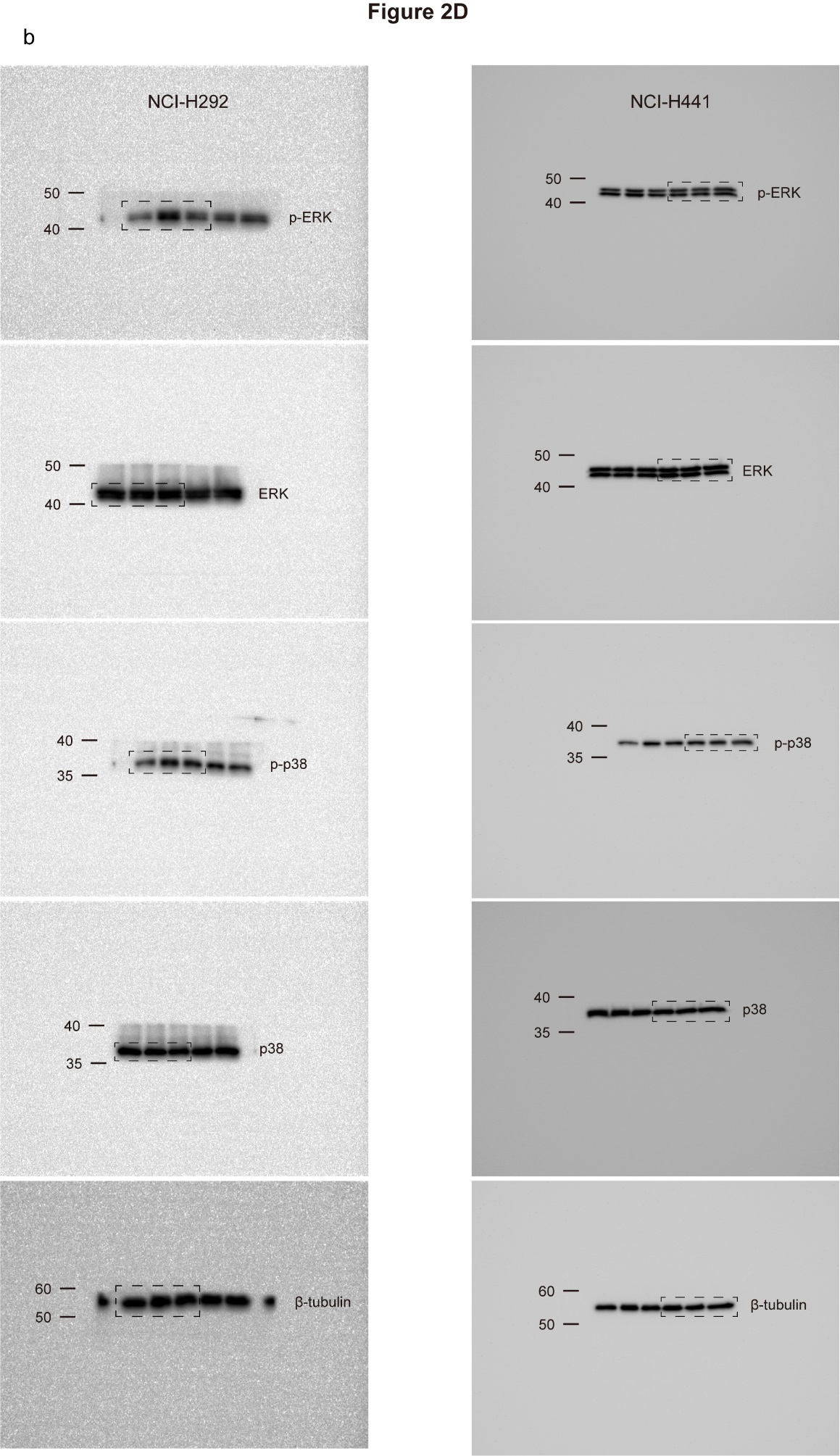


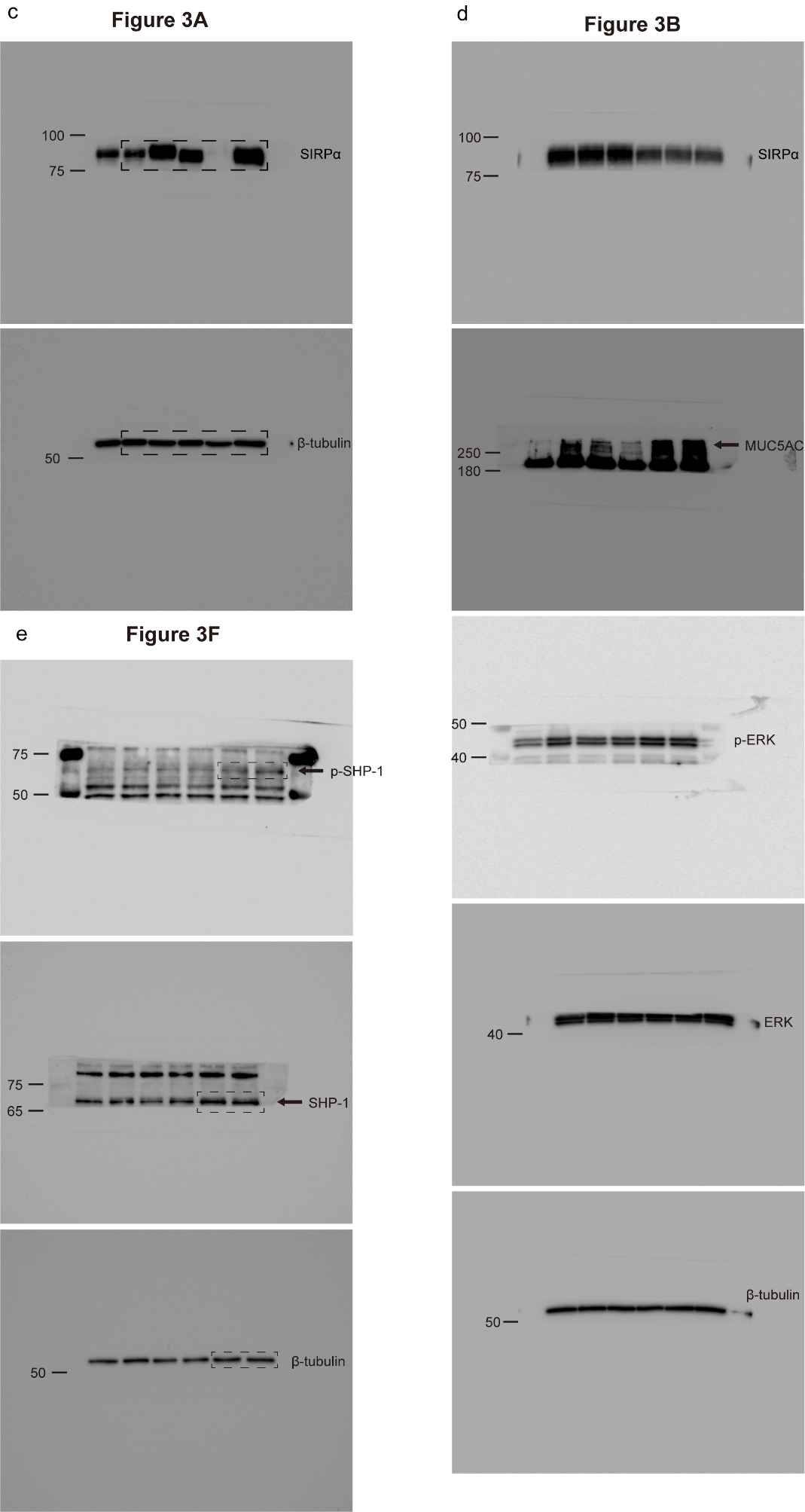


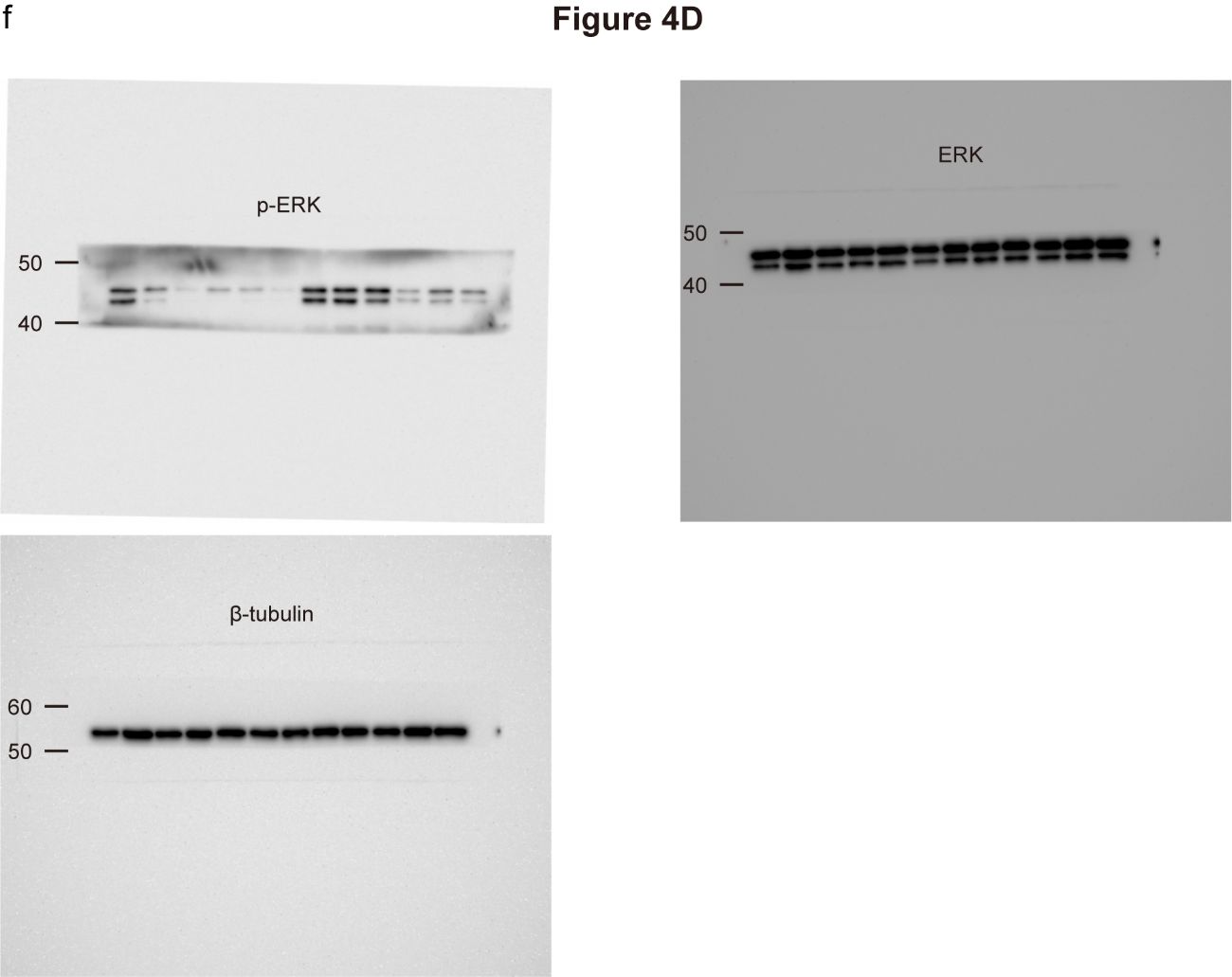


**Long and short exposure images from Western blots.**

Short exposure and long exposure Western blot images are shown that correspond to Figure 2B (a), Figure 2D (b), Figure 3A (c), Figure 3B (d), Figure 3F (e), and Figure 4D (f).


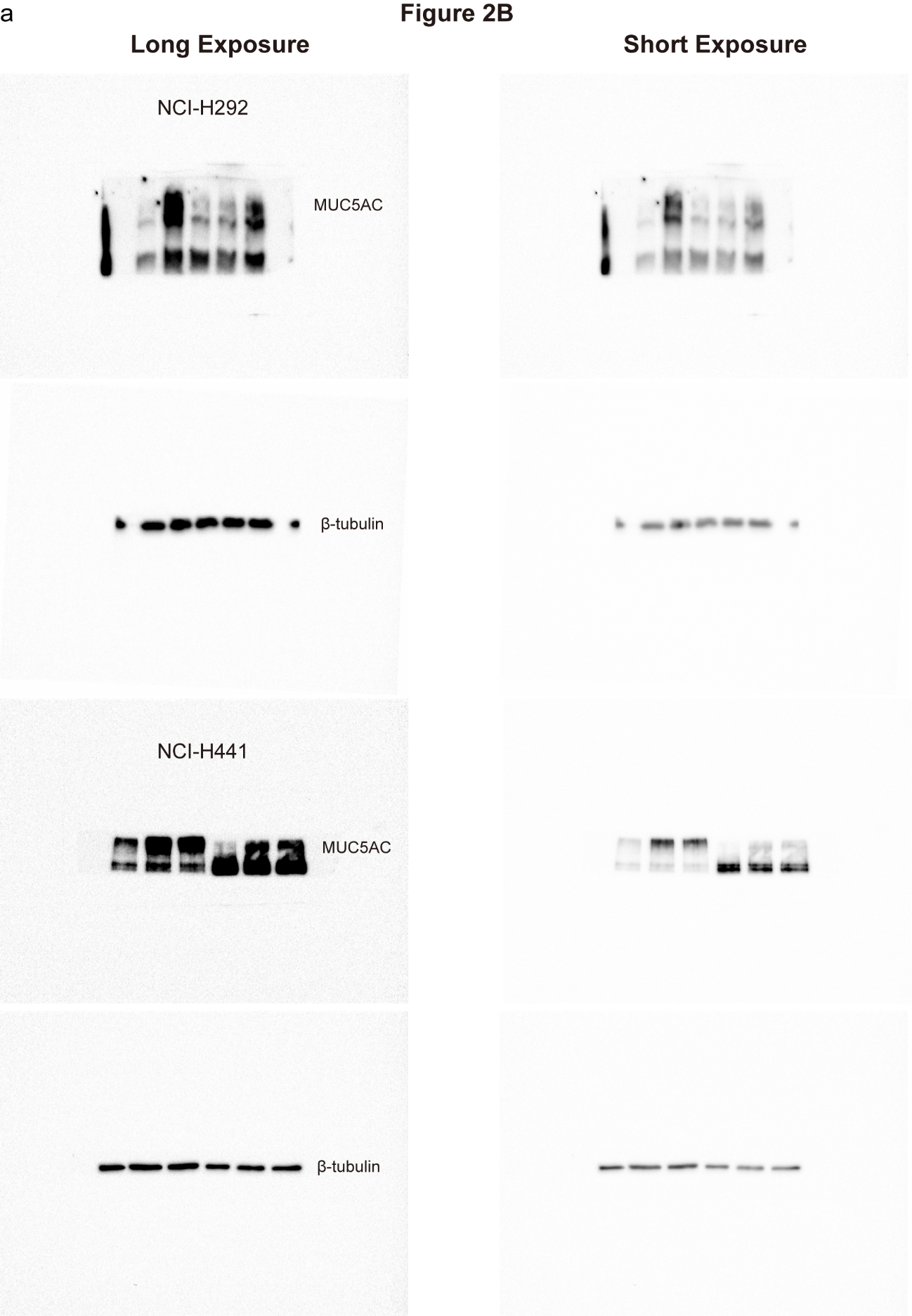


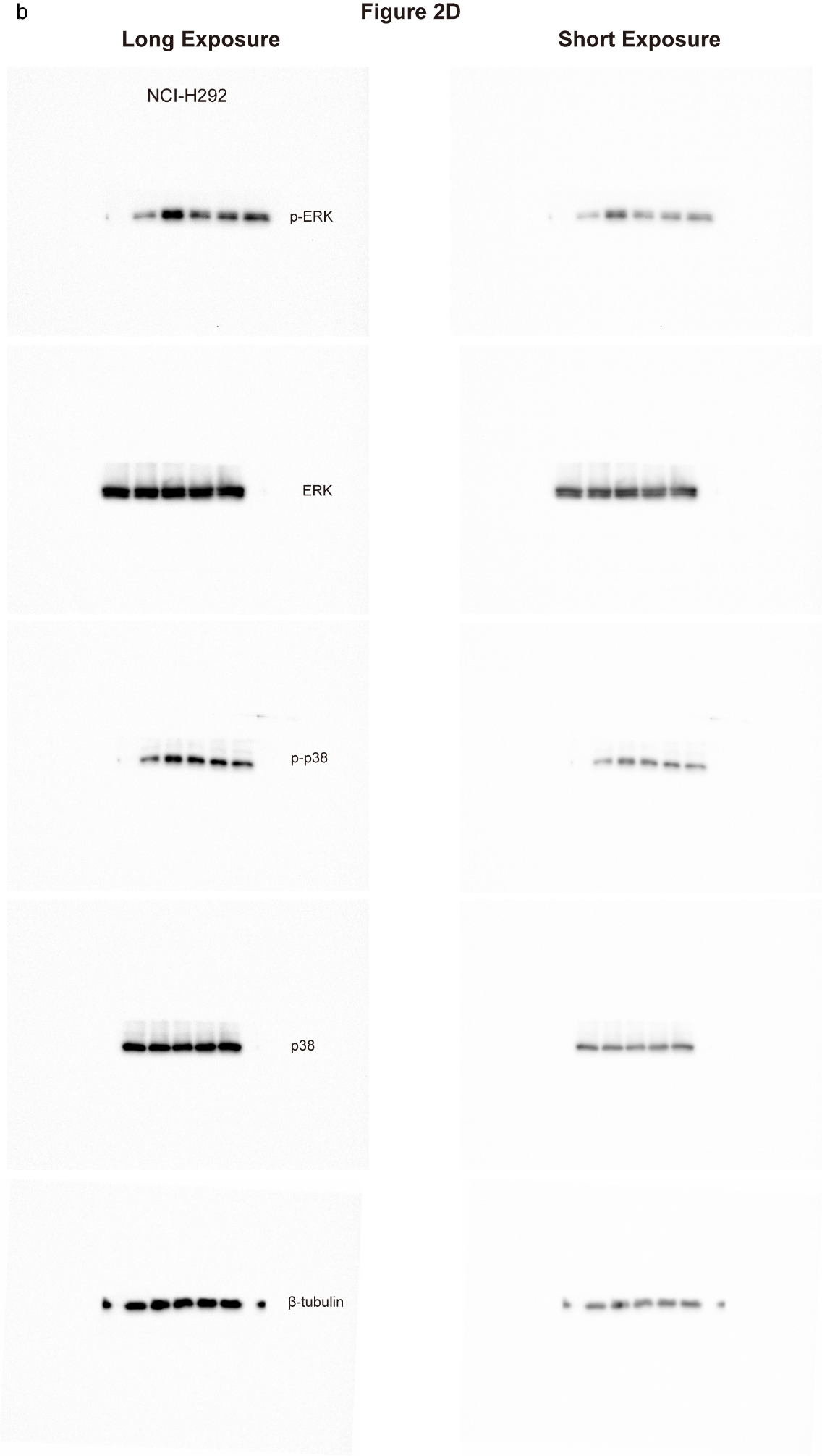


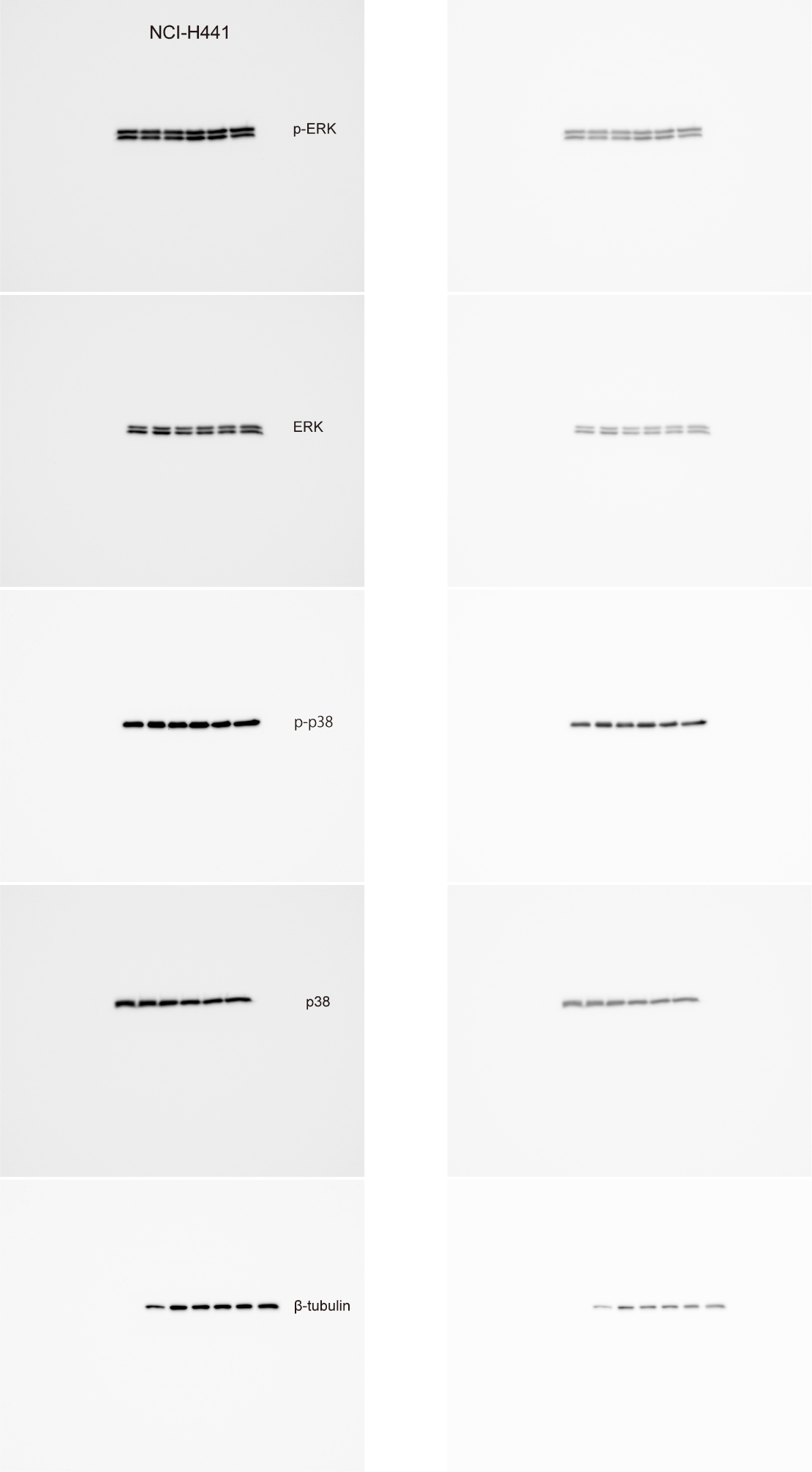


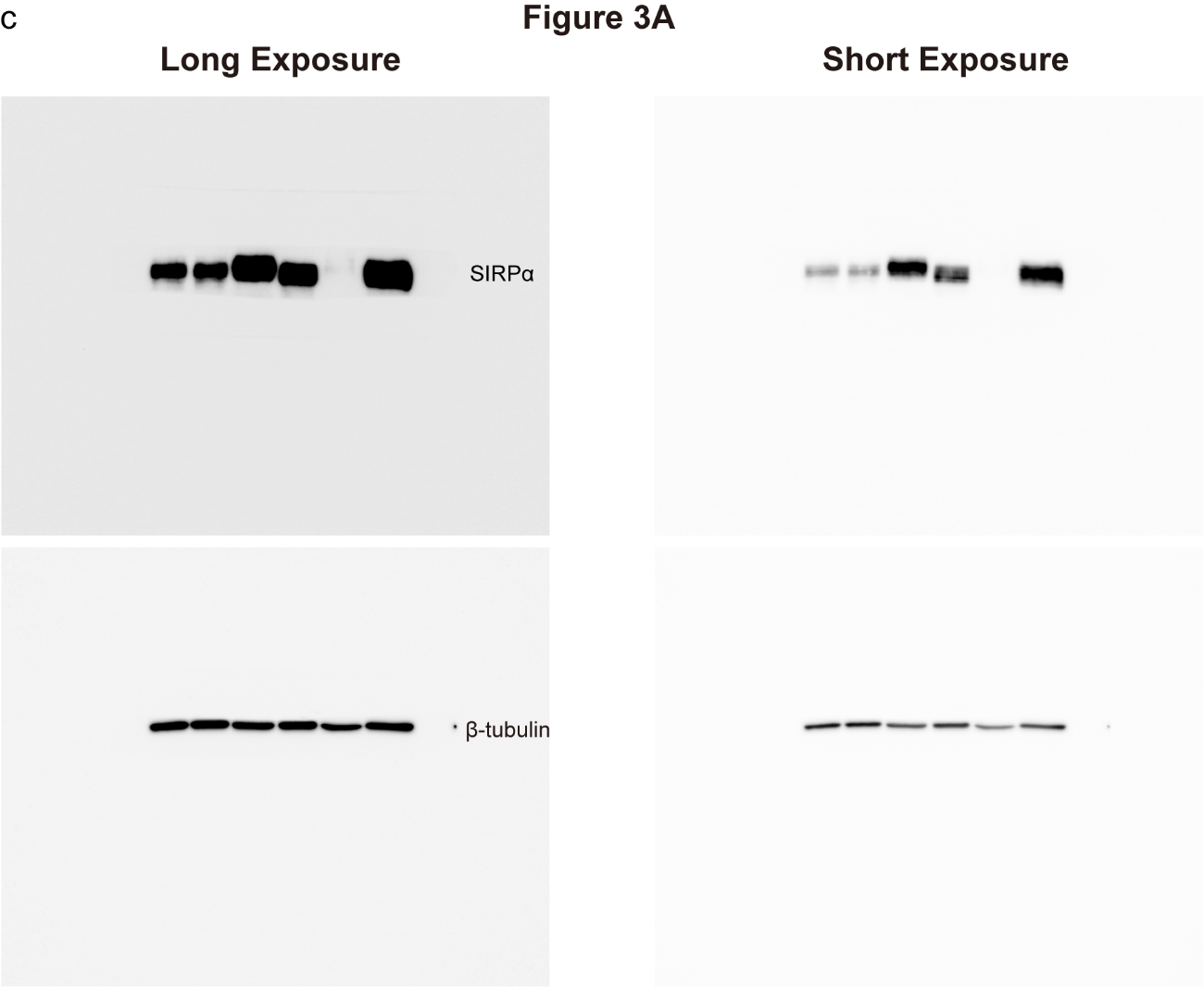


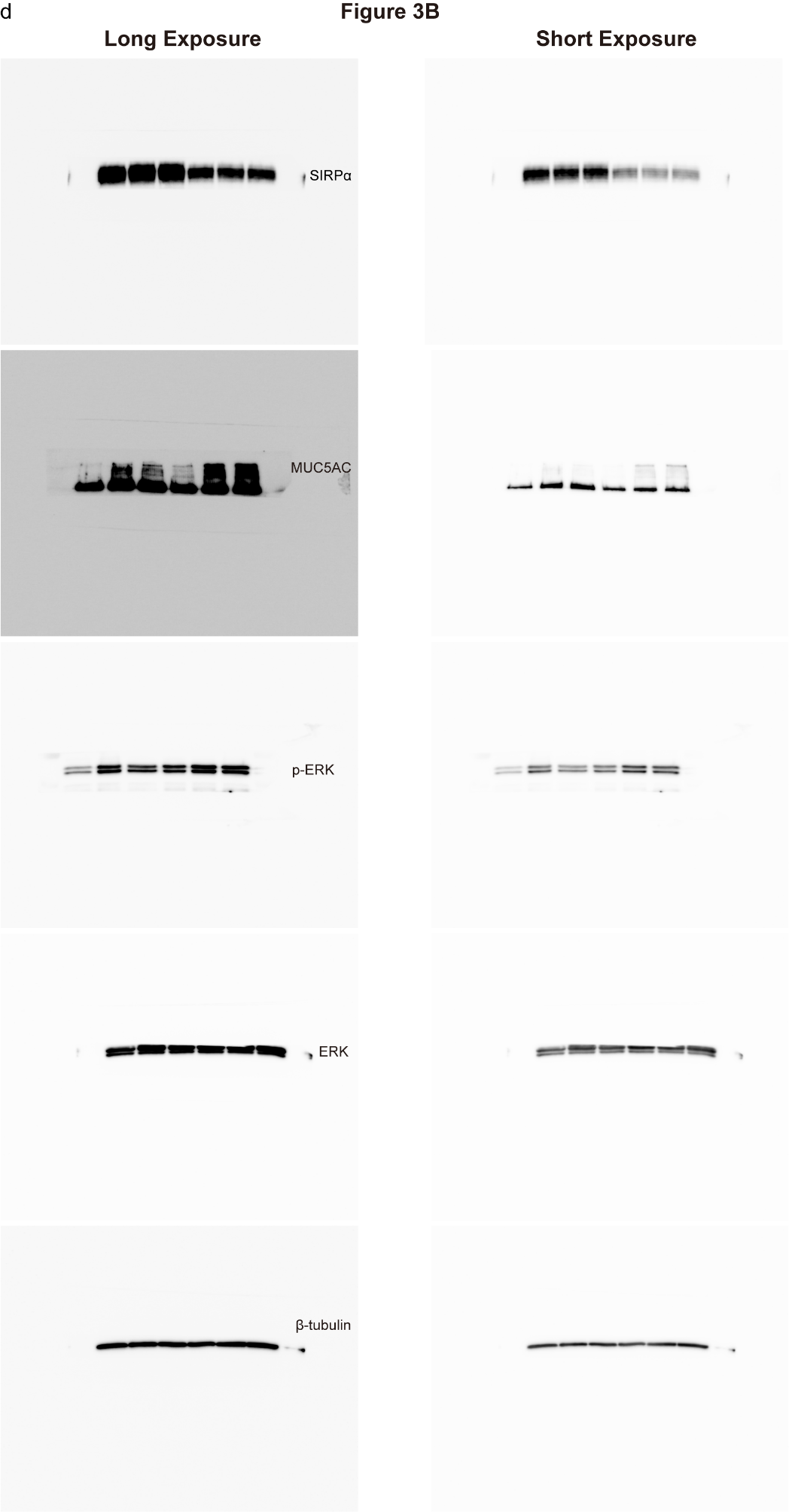


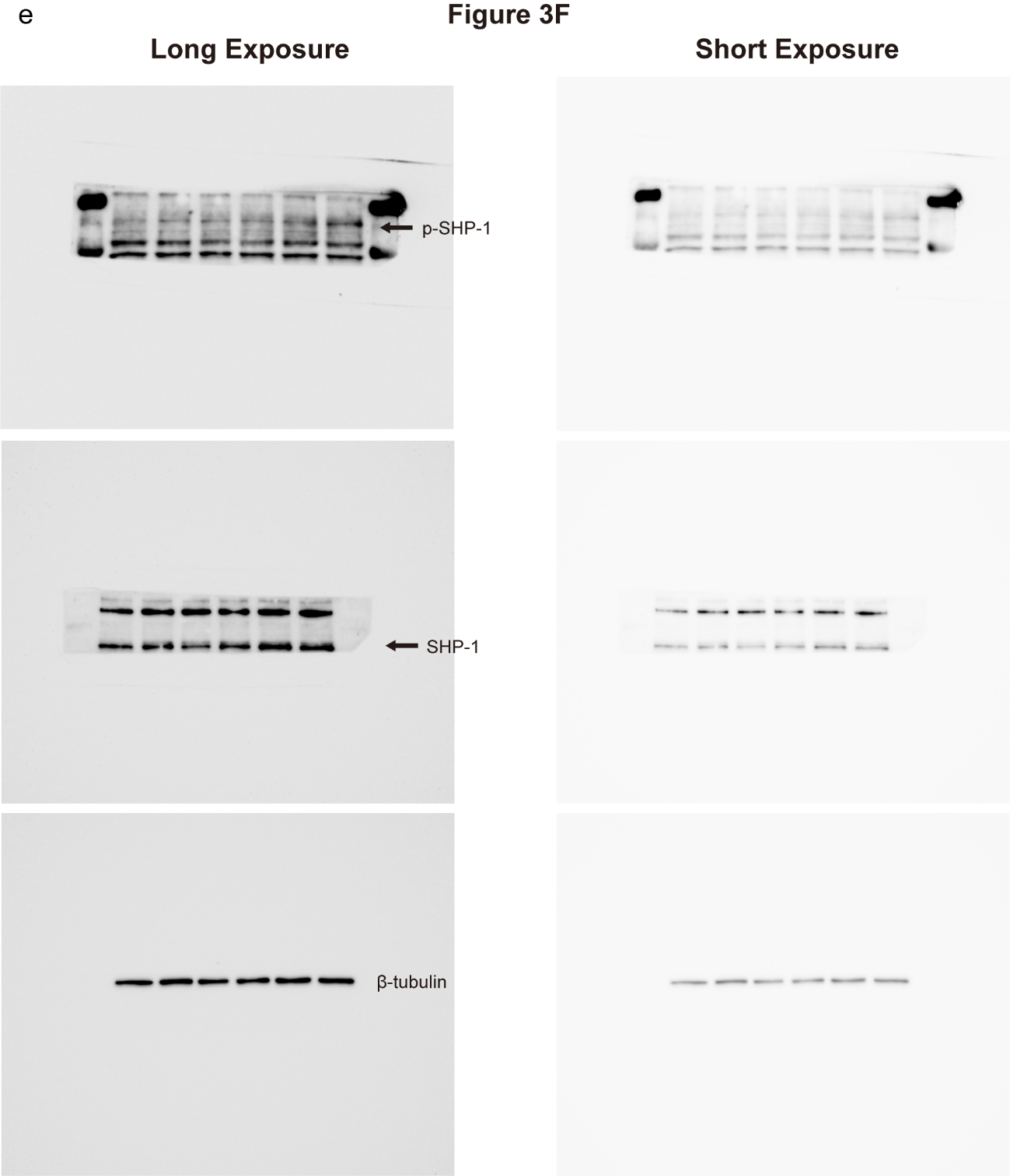


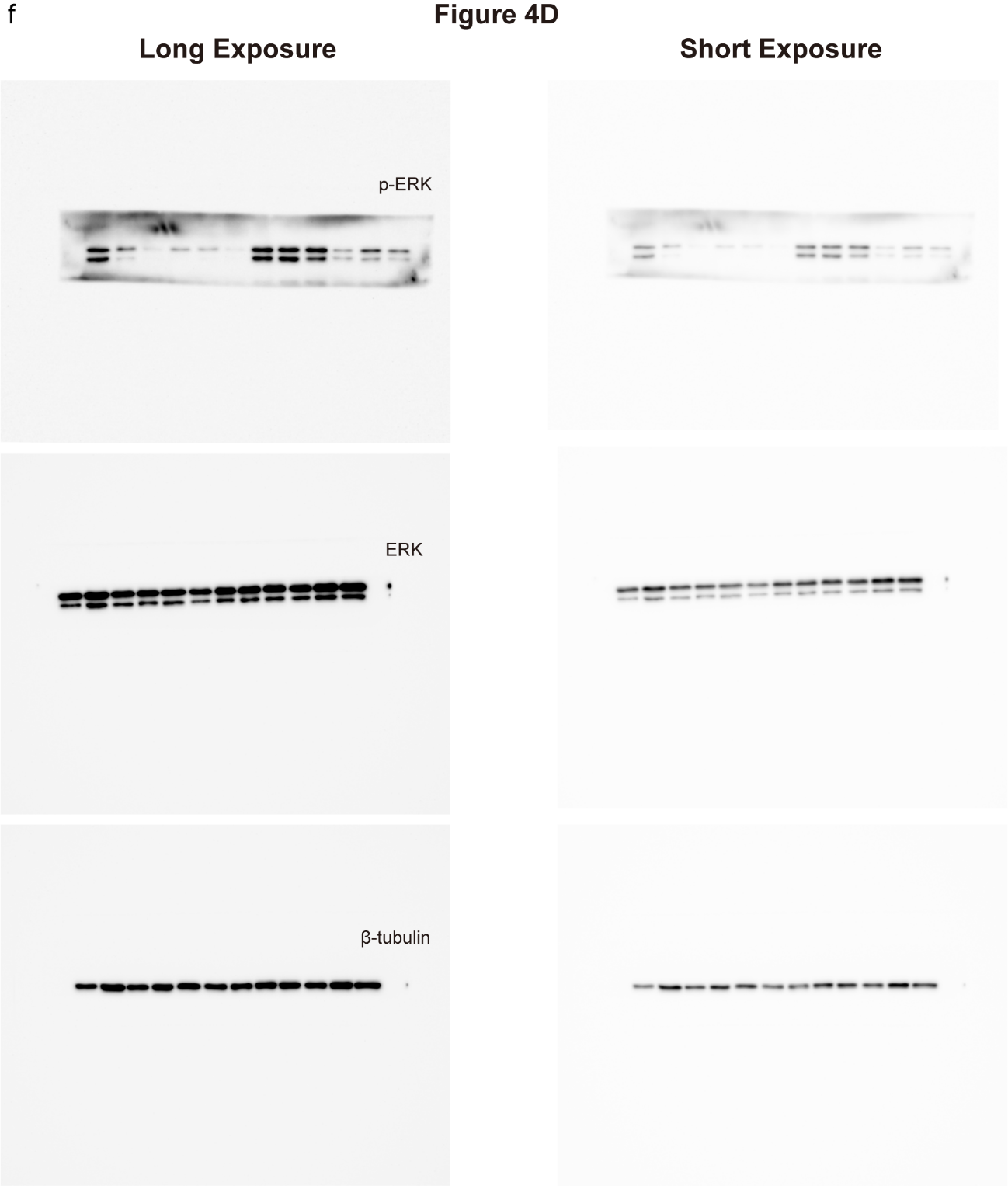

Supplement: Supplementary file 2 — Supplementary Information 2. [file 41598_2024_52328_MOESM2_ESM.docx]
